# Prognostic signature for hepatocellular carcinoma based on 4 pyroptosis-related genes

**DOI:** 10.1186/s12920-022-01322-9

**Published:** 2022-07-28

**Authors:** Sainan Duan, Jianying Gao, Weiming Lou, Yize Zhang, Ying Deng, Cong Wang, Haiyue Huang, Hui Xu, Sixuan Guo, Shuhui Lai, Feiyang Xi, Zhangwang Li, Libin Deng, Yuanbin Zhong

**Affiliations:** 1 The First Affiliated Hospital of Nanchang University, Nanchang University, Nanchang, Jiangxi Province China; 2 Jiangxi Provincial Key Laboratory of Preventive Medicine, School of Public Health, Nanchang University, Nanchang, 330006 Jiangxi China; 3 Institute of Translational Medicine, Nanchang University, Nanchang, China; 4 The Second Affiliated Hospital of Nanchang University, Nanchang University, Nanchang, Jiangxi Province China; 5 Queen Mary School of Nanchang University, 999 Xuefu Road, Nanchang, 330031 Jiangxi China; 6 The Public Health College of Nanchang University, Nanchang, China; 7 College of Basic Medical, Nanchang University, Nanchang, 330006 Jiangxi China; 8 Department of Infectious Diseases and Key Laboratory of Liver Regenerative Medicine of Jiangxi Province, The First Affiliated Hospital of Nanchang University, Nanchang, 330006 Jiangxi China

**Keywords:** Hepatocellular carcinoma, Pyroptosis, Risk scores

## Abstract

**Background:**

Hepatocellular carcinoma (HCC) is a cancer with a poor prognosis. Many recent studies have suggested that pyroptosis is important in tumour progression. However, the role of pyroptosis-related genes (PRGs) in HCC remains unclear.

**Materials and methods:**

We identified differentially expressed PRGs in tumours versus normal tissues. Through univariate, LASSO, and multivariate Cox regression analyses, a prognostic PRG signature was established. The signature effectiveness was evaluated by time-dependent receiver operating characteristic (t-ROC) curve and Kaplan–Meier (KM) survival analysis. The signature was validated in the ICGC (LIRI-JP) cohort. In addition, single-sample gene enrichment analysis (ssGSEA) showed the infiltration of major immune cell types and the activity of common immune pathways in different subgroups.

**Results:**

Twenty-nine pyroptosis-related DEGs from The Cancer Genome Atlas-Liver Hepatocellular Carcinoma (TCGA-LIHC) dataset were detected, and four genes (*CTSV*, *CXCL8*, *MKI67* and *PRF1*) among them were selected to construct a prognostic signature. Then, the patients were divided into high- and low-risk groups. The pyroptosis-related signature was significantly associated with overall survival (OS). In addition, the patients in the high-risk group had lower levels of immune infiltration.

**Conclusion:**

The prognostic signature for HCC based on 4 pyroptosis-related genes has reliable prognostic and predictive value for HCC patients.

**Supplementary Information:**

The online version contains supplementary material available at 10.1186/s12920-022-01322-9.

## Background

Hepatocellular carcinoma (HCC) is one of the most common cancers, and its outlook is very poor. Worldwide, it is a leading cancer type in 11 countries and regions and is the third leading cause of cancer-related death in 23 countries after lung cancer and colorectal cancer. According to GLOBCAN 2020, 830,180 new deaths from HCC occur every year [1].In most patients, surgery is only an option for those in the early stages of liver cancer (less than stage IIIB and Child–Pugh stage A) [2]. Patients with advanced liver cancer often take conservative treatments and have a poor prognosis. However, the early clinical manifestations of HCC are often atypical, and it is difficult to diagnose HCC only from the clinical manifestations. AFP is a common serum molecular marker used to diagnose liver cancer. However, normal AFP levels may be present in approximately 30% of patients with HCC [3]. Accordingly, the establishment of a multigene classifier to predict HCC outcomes is important. Such a classifier could have important reference value for predicting the survival and treatment outcomes of patients with liver cancer.

Pyroptosis is a form of regulated cell death (RCD), and the Nomenclature Committee on Cell Death recommended that it be recognized as a type of RCD that critically depends on the formation of plasma membrane pores by members of the gasdermin (GSDM) protein family, often (but not always) as a consequence of inflammatory caspase activation [4]. GSDM pores formed in the plasma membrane eventually lead to cell lysis, and a large number of damage-related molecular patterns (DAMPs), such as ATP, IL-1β, and HMGB1, are released at different times and stages [5], resulting in inflammation. Studies have shown that certain inflammatory factors, such as IL-1β, play a role in promoting tumour invasion. Solid tumours that have been shown to upregulate IL-1β include breast, colon, lung, and head and neck cancers and melanoma, and patients with these IL-1β-upregulated tumours generally show a poor prognosis [6]. In many tumours, IL-1β may increase tumour cell metastasis by promoting angiogenesis [7, 8]. However, IL-1β has also been shown to inhibit cancer. Haabeth et al. demonstrated that IL-1β can drive tumour-specific Th1 response activation to inhibit the progression of B-cell myeloma and lymphoma [9, 10]. However, there are few studies on pyroptosis in HCC.

Here, we conducted a systematic study of pyroptosis-related genes (PRGs) in HCC to determine differences in the expression of these genes between tumour patients and nontumour patients, to assess the correlation between their expression and patient prognosis and to study the effects of these PRGs on the tumour-immune microenvironment.

## Materials and methods

### Data collection

The mRNA expression profiles (mRNA-seq) and corresponding clinical data of patients were obtained from The Cancer Genome Atlas (TCGA) database. We downloaded TCGA-liver hepatocellular cancer (LIHC) sample information after log2 (FPKM + 1) processing from the Xena platform (https://xena.ucsc.edu/) [11]. Our inclusion criteria for patients were as follows: histologically diagnosed with hepatocellular carcinoma [12]; available expression profiles; patients with survival information. Consequently, we extracted 267 tumour samples from the TCGA dataset for subsequent analysis (Table 1). Under the same criteria, 240 primary tumour solid tissue samples (Table 2) were included as the external validation dataset from the International Cancer Genome Consortium (ICGC), and 184 PRGs were retrieved from the GeneCards database (25th August 2021. https://www.genecards.org/) with the keyword “pyroptosis”. The above three databases are publicly available and accessible.

**Table 1:** Clinicopathologic characteristics of HCC patients in TCGA database

Characteristic	Levels	Overall
n		367 (100%)
Gender	Female	119 (32.43%)
	Male	248 (67.57%)
Age	≤ 65	230 (62.67%)
	> 65	137 (37.33%)
Stage	Stage I	171 (46.59%)
	Stage II	85 (23.16%)
	Stage III	83 (22.62%)
	Stage IV	4 (1.09%)
	Unknown	14 (3.81%)
Grade	G1	55 (14.99%)
	G2	176 (47.96%)
	G3	119 (32.43%)
	G4	12 (3.27%)
	Unknown	5 (1.36%)

Clinical information on HCC samples in the TCGA database includes gender, age, stage and grade

**Table 2:** Clinicopathologic characteristics of HCC patients in ICGC database

Characteristic	Levels	Overall
n		240 (100%)
Gender	Female	61 (25.42%)
	Male	179 (74.58%)
Age	≤ 65	155 (64.58%)
	> 65	85 (35.42%)
Stage	Stage I	36 (15%)
	Stage II	109 (45.42%)
	Stage III	74 (30.83%)
	Stage IV	21 (8.75%)

Clinical information on HCC samples in the ICGC database includes gender, age, stage

### Consensus clustering (CC) analysis

Unsupervised class discovery is a highly useful technique in cancer research, where intrinsic groups sharing biological characteristics may exist but are unknown. The CC method provides quantitative and visual stability evidence for estimating the number of unsupervised classes in a dataset [13]. We used the “ConsensusClusterPlus” R package (version 1.52.0) to duplicate tumour samples in TCGA 1000 times to find the optimal classification cluster of tumour samples, and approximately 80% of the samples were selected in each iteration.

### Search for differentially expressed genes (DEGs) in the TCGA-LIHC dataset

We used the empirical Bayesian approach of the “limma” R package (version 3.44.3) to obtain DEGs [14]. DEGs with |log2-fold change (FC)|≥ 1 and adj *P*-value (FDR) < 0.05 were used for subsequent analysis.

### Construction of the prognostic PRG signature

Univariate Cox regression, least absolute shrinkage and selection operator (LASSO)-Cox regression and multivariate Cox regression analyses were used to identify the genes associated with pyroptosis and establish a prognostic PRG signature for HCC. In the univariate Cox regression analysis, *P* < 0.05 was considered to indicate statistical significance, indicating that the gene is associated with overall survival (OS). The LASSO Cox regression model was then utilized to narrow down the candidate genes by using tenfold cross validation via the “glmnet” R package [15]. Then, we carried out multivariate Cox regression analysis and established the risk scoring equation according to the standardized regression coefficients of the factors.

$${\text{Risk Score}} = \Sigma {\text{i}}({\text{Coefi}} \cdot {\text{Expi}})$$

To evaluate the predictive ability of the prognostic PRG signature, we used the median risk score to divide the sample into high- and low-risk groups. Kaplan–Meier (KM) survival curves were drawn with the "survival" R package (version 3.2-13), and the *P* value was calculated. In addition, time-dependent receiver operating characteristic (t-ROC) curve analyses were performed to determine how accurately the signature predicted patient survival at six months, one year, two years, and three years [16]. The risk curve was also used to verify the relationship between the risk score and patient survival status. Principal component analysis (PCA) integrated the risk score and patient survival information through dimensionality reduction and presented relevant information in a low-dimensional manner, which could verify the signature's ability to distinguish between high- and low-risk groups [17]. In addition, we performed a similar process with data from the ICGC database, and the prognostic PRG signature was further validated in this independent cohort.

### Validation of prognostic genes in an external database

The mRNA expression levels of four marker genes were verified with the Tumour Immune Estimation Resource (TIMER) database (https://cistrome.shinyapps.io/timer/). In addition, we used the cBioPortal database (https://www.cbioportal.org/) to investigate the genetic alterations of the related genes. The above two databases are publicly available and accessible.

### Construction of the nomogram and evaluation of its predictive value

The expression levels of the four marker genes in the risk scoring equation were included in the nomogram to assess the 1-year, 2-year and 3-year survival rates of the patients, and calibration curves were drawn to assess the prognostic performance of the nomogram.

### Validation of the independence of the risk scores from other clinical variables

The clinical information, including sex, age, tumour grade, tumour stage and risk scores, of patients providing the samples was included in the univariate Cox regression analysis, and bilateral *P* values < 0.05 were considered to be statistically significant. The clinical features were also incorporated into the multivariate Cox regression analysis to determine whether the prognostic model prediction ability was independent of the conventional clinical features. Hazard ratios (HRs) and 95% confidence intervals (CIs) were calculated.

### Gene enrichment analysis

We next investigated the biological processes involving the DEGs between the high- and low-risk patient groups. DEGs with *P* < 0.05 and absolute value of log2 FC > 1 were selected for enrichment analysis. For the Kyoto Encyclopedia of Genes and Genomes (KEGG) [18–20] and gene ontology (GO) analyses, *P* < 0.05 and false discovery rate (q) < 1 were considered indicators of statistical significance.

### Immune infiltration analysis

The "GSVA" package (version 1.32.3) was used to calculate the immune score of the samples, explore the infiltration of 16 major immune cell types and investigate the activity of 13 common immune pathways in different subgroups in the high- and low-risk groups.

### Statistical analysis

**Fig. 1:**
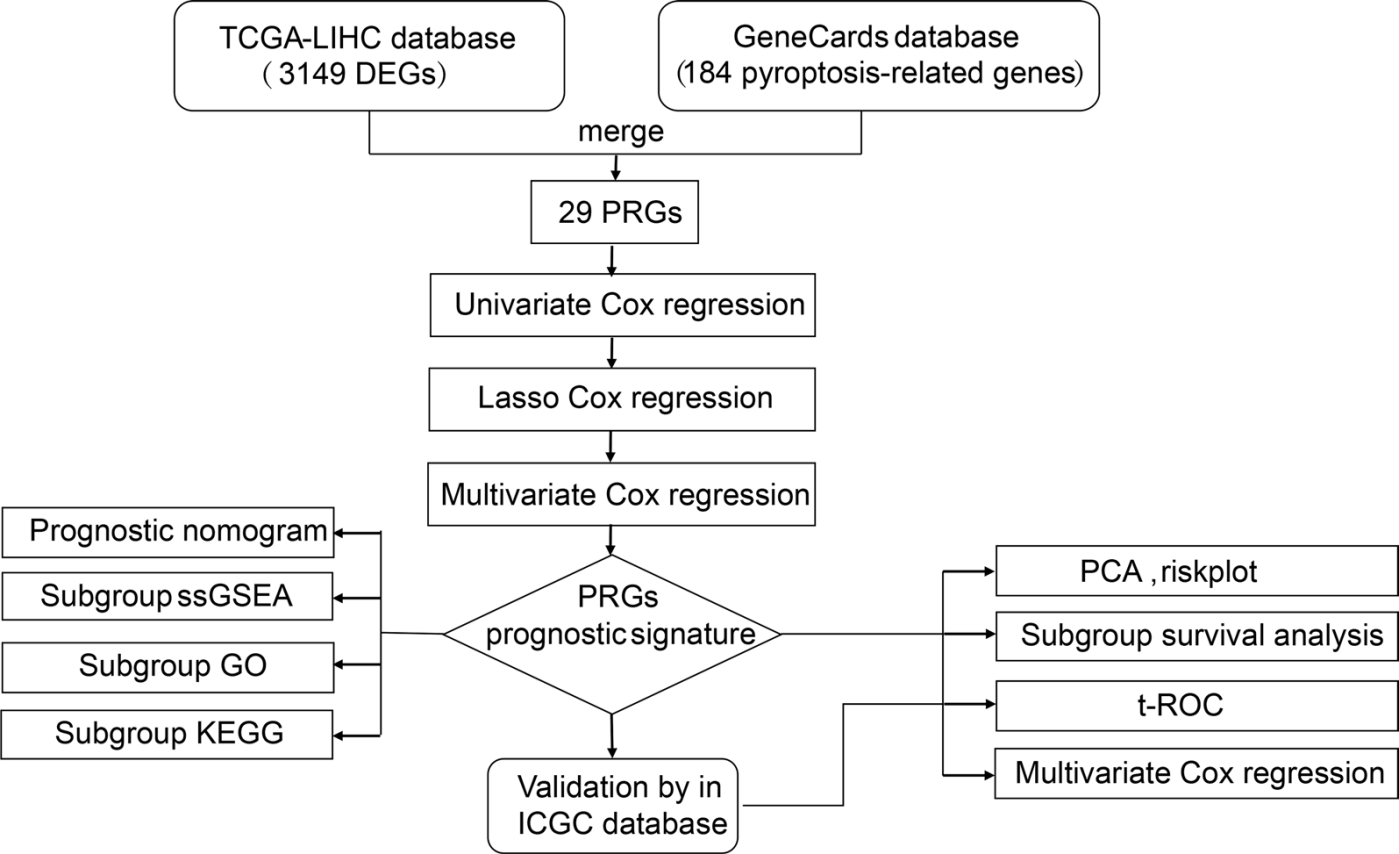
The flowchart of the whole study. Flowchart for establishing and verifying prognostic signature

Single-factor analysis of variance was applied to compare the gene expression of 184 PRGs between the normal and HCC tissues. Independent prognostic factors were identified by univariate and multivariate Cox regression. Differences in OS between high- and low-risk groups were assessed using Kaplan–Meier analysis and two-sided log-rank test. When comparing the immune cell infiltration and immune pathway activation between the two groups, the Mann–Whitney test was used. All statistical *P* values are two-side and *P* < 0.05 represents statistical significance. Besides, R software (Version 4.0.2) was employed for all statistical data analyses. The flowchart of the whole study is shown in Fig. 1.

## Results

### CC analysis to explore patient differences

To effectively distinguish liver cancer patients by PRGs, we conducted CC analysis on all HCC patients in the TCGA cohort. The cluster variable K was increased from 2 to 10; when K = 2, the correlation within subgroups was the highest, while the correlation between groups was the lowest. However, the samples could not be well distinguished (Additional file 1: Fig. S1a). In addition, when KM survival curves were generated for the two subgroups, we found no significant difference between the two groups (Additional file 1: Fig. S1b).

### Identification of differentially expressed PRGs between tumour and normal tissue

**Fig. 2:**
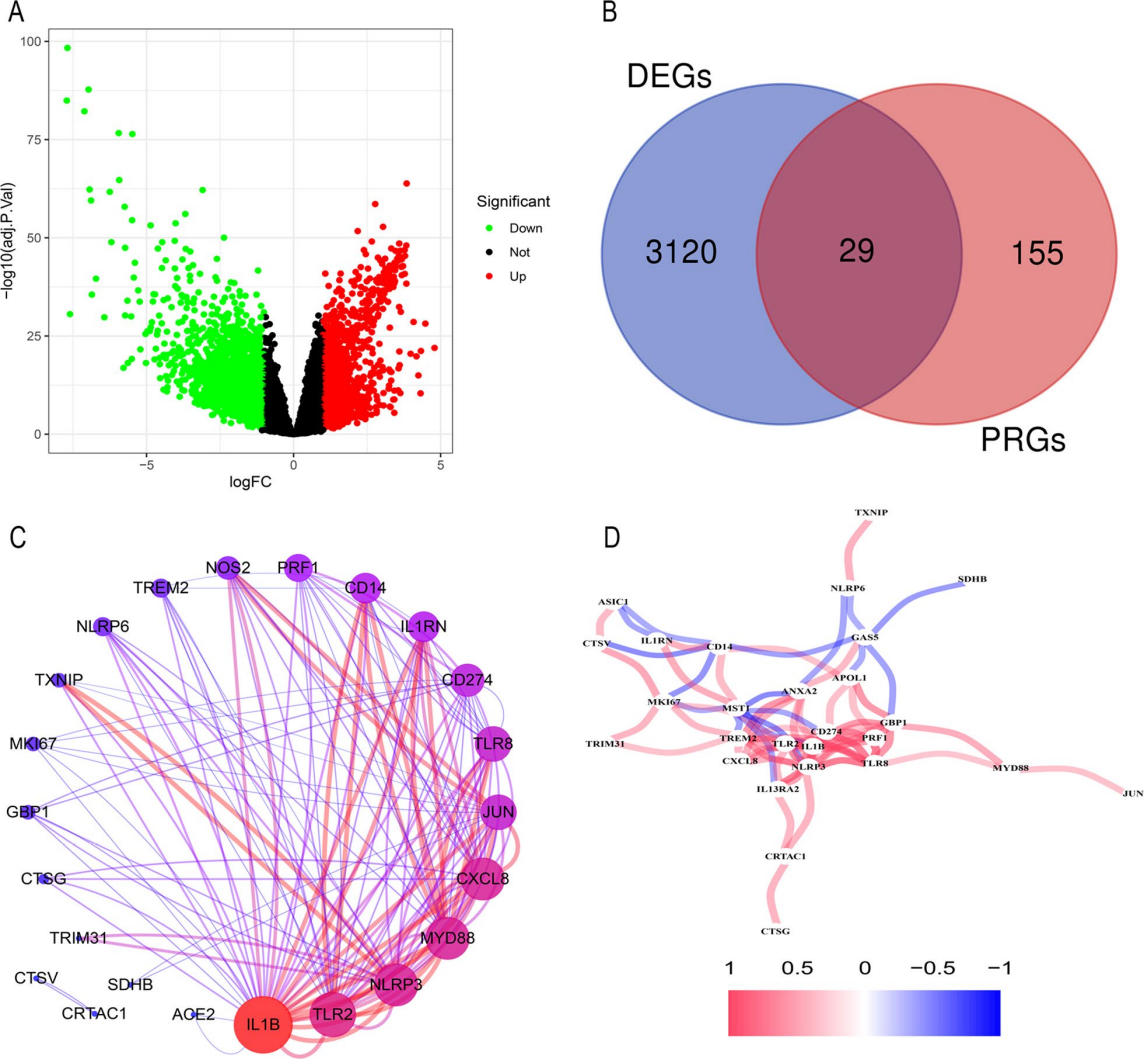
Searching for PRGs and links between them. Volcano plot of all DEGs between HCC and normal samples. Red: upregulated DEGs; Black: nonsignificant genes; Green: downregulated DEGs (**a**). Overall, 29 PRGs were found in DEGs (**b**). PPI analysis and the correlation network were drawn to explore the relationship between 29 genes (**c**, **d**)

Overall, 184 genes related to pyroptosis were retrieved from the GeneCards database, and 29 differentially expressed genes with |Log2 FC|> 1 were screened by the "limma" package (Fig. 2a,b). *P* < 0.05 was considered to indicate statistically significant DEGs. Of the DEGs, 8 PRGs were upregulated in liver cancer tissues, and 21 PRGs were downregulated in tumour tissues.

To further explore the association between these PRGs, protein–protein interaction (PPI) analysis was performed, setting the minimum required interaction score at 0.4 (medium confidence). Cytoscape software (Version 3.7.2) was employed for visualization (Fig. 2c), and we found that IL1B interacts with many genes at the protein level. In addition, we mapped the correlation network containing these 29 genes and explored their relationships at the gene level (Fig. 2d).

### Construction and development of a prognostic signature for HCC

**Fig. 3:**
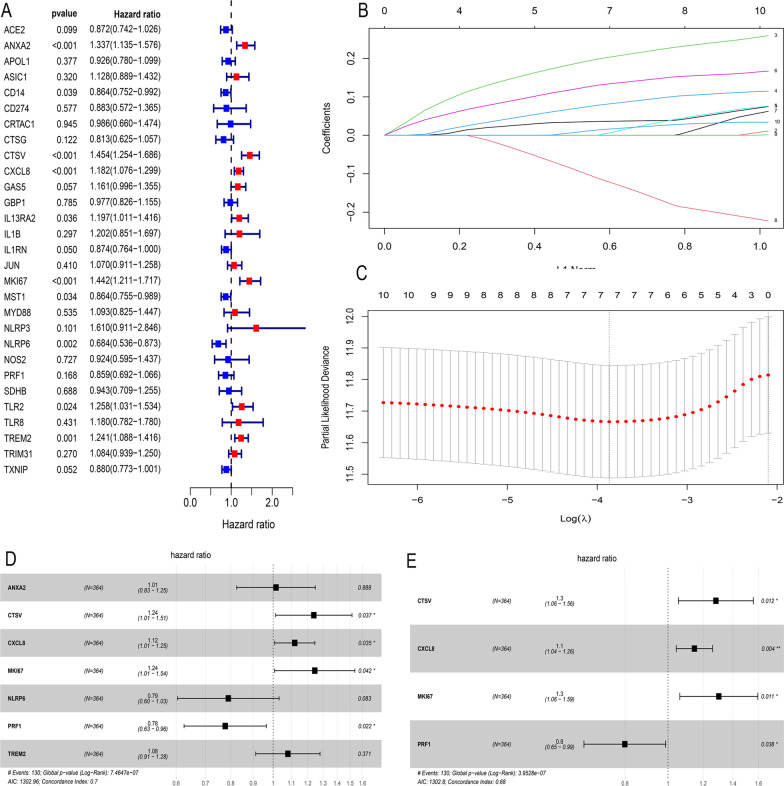
Construction and development of a prognostic signature for HCC. Univariate Cox regression was used to identify genes associated with OS (**a**). LASSO regression of the 10 OS-related genes (**b**, **c**). The prognostic signature was established based on multivariate Cox regression (**d**, **e**)

**Fig. 4:**
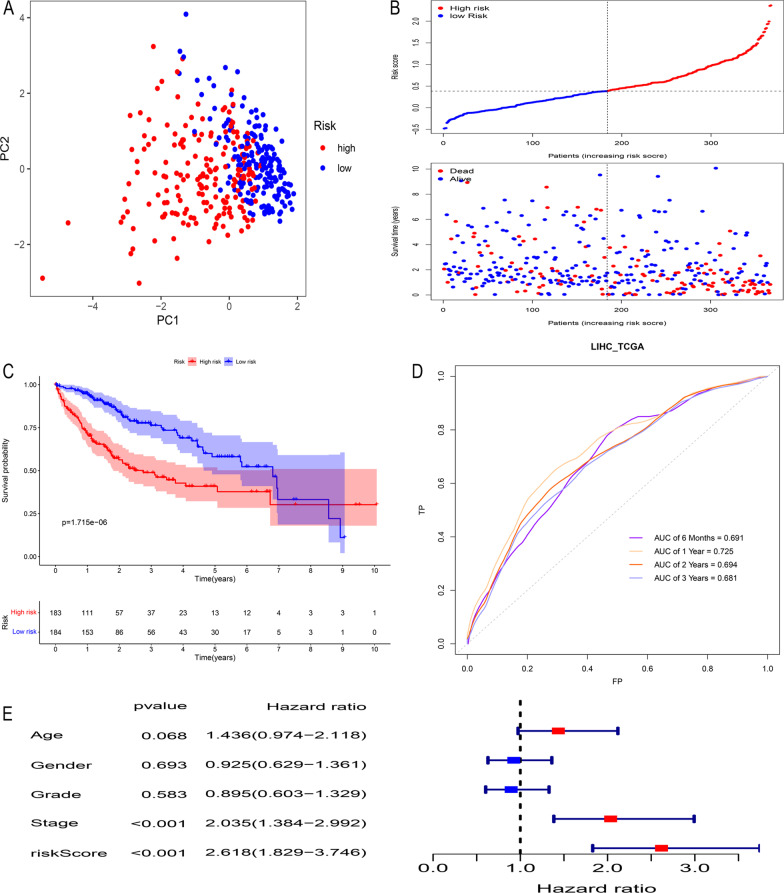
Internal validation of the TCGA cohort. PCA plot for samples based on the risk scores (**a**). Risk score and survival status distribution of HCC samples from the TCGA database (**b**). KM curves of samples in the high- and low-risk groups (**c**). t-ROC curves demonstrated the predictive efficiency of the risk score (**d**). Multivariate Cox regression showed that the risk score was an independent prognostic factor in the TCGA database (**e**)

Through univariate Cox regression analysis, we found 10 DEGs related to the prognosis of HCC among 29 PRGs (Fig. 3a). High expression of *ANXA2*, *CTSV*, *CXCL8*, *IL13RA2*, *MKI67*, *TLR2* and *TREM2* may promote survival, while high expression of *CD14*, *MST1* and *NLRP6* may adversely affect survival. LASSO regression analysis further narrowed ten genes down to seven (*ANXA2*, *CTSV*, *CXCL8*, *MKI67*, *NLRP6*, *NLRP6*, and *PRF1*) (Fig. 3b, c). Finally, we obtained 4 key prognosis-related PRGs—*CTSV*, *CXCL8*, *MKI67* and *PRF1*—by stepwise multivariate regression analysis (Fig. 3d, e). The prognostic PRG signature was constructed as follows: risk score = (0.249* *CTSV* expression) + (0.137* *CXCL8* expression) + (0.264* *MKI67* expression) + (− 0.224* *PRF1* expression). *CTSV*, *CXCL8* and *MKI67* were high-risk factors (HR > 1), and *PRF1* was a protective factor (HR > 1). After calculating the risk scores of all samples, the median was used to divide the samples into two subgroups (high and low risk), with a cut-off value of 0.384. Patients in the high- and low-risk groups were well distinguished by PCA (Fig. 4a), and the high-risk score group had a higher mortality rate and shorter survival time (Fig. 4b, c). By plotting t-ROC curves, we found that risk scores also had certain significance in differentiating patients’ half-year, 1-year, 2-year, and 3-year survival (Fig. 4d). The area under the t-ROC curve (AUC) was 0.691 at 6 months, 0.725 at 1 year, 0.694 at 2 years and 0.681 at 3 years. In addition, multivariate Cox regression proved that our prognostic signature was an independent prognostic factor for HCC (Fig. 4e).

### External database validation of the PRG-related prognostic signature in HCC

**Fig. 5:**
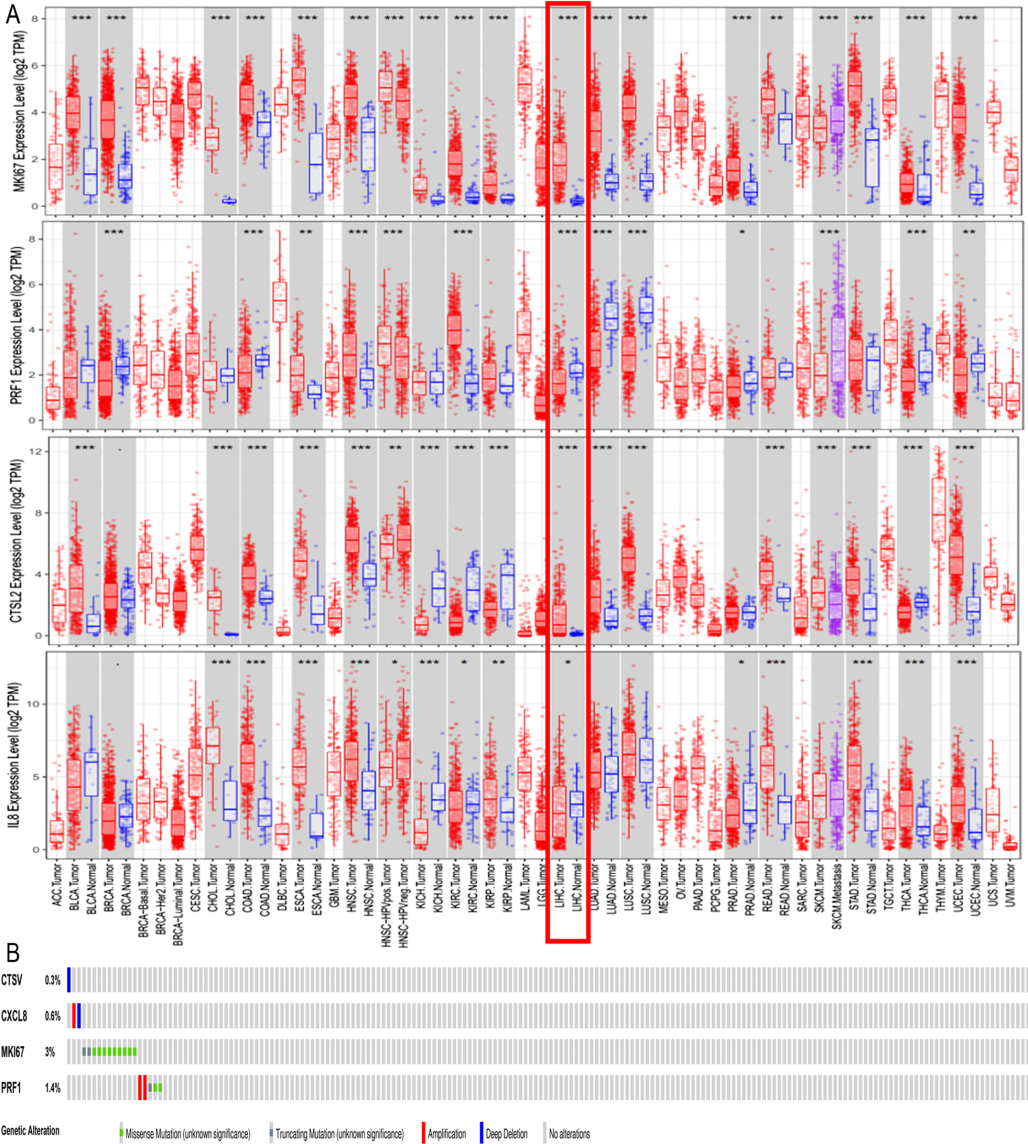
External database validation of the PRG-related prognostic signature in HCC. Human CTSV, CXCL8, MKI67 and PRF1 levels in different tumour types from TCGA were determined using TIMER (**a**). The mutations of these four genes in TCGA HCC samples were assessed via the cBioPortal database (**b**)

TIMER database analysis confirmed that *CTSV* and *MKI67* were highly expressed in tumour tissues, while *CXCL8* and *PRF1* were expressed at low levels in tumour tissues (Fig. 5a), which was consistent with our findings. We investigated the genetic changes in the *CTSV*, *CXCL8*, *MKI67* and *PRF1* genes. The mutations of these four genes in TCGA HCC samples were assessed via the cBioPortal database. *MKI67* had the highest frequency of genetic alterations (3%), and missense mutation was the most common alteration (Fig. 5b).

### Construction of a nomogram

**Fig. 6:**
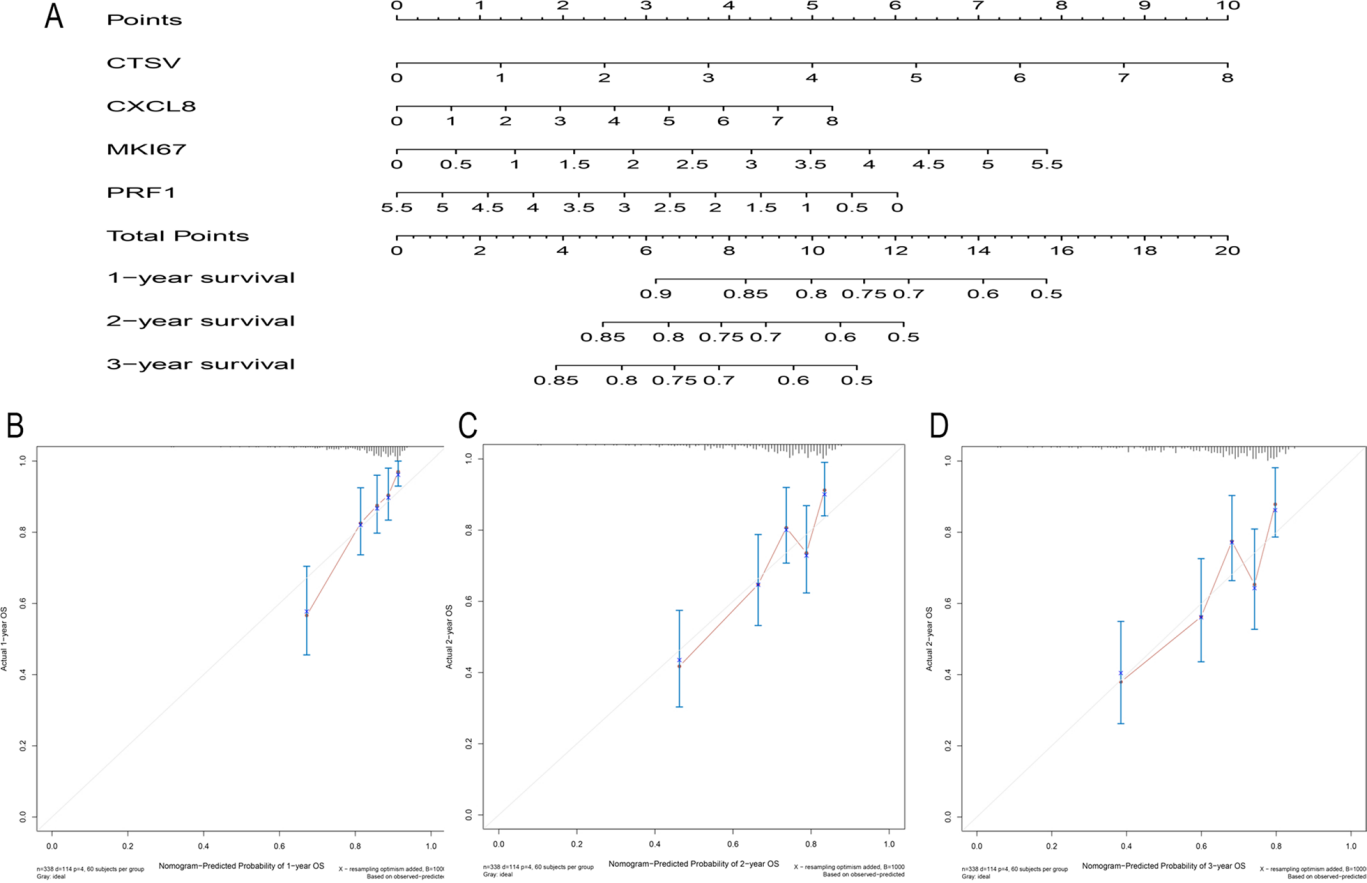
Construction and verification of a nomogram. The nomogram was built based on the expression of 4 genes in the training cohort (**a**). The calibration plots showed good predictive performance for OS at 1, 2, and 3 years (**b**–**d**)

Univariate and multivariate Cox regression analyses showed that the expression of *CTSV*, *CXCL8*, *MKI67* and *PRF1* were independent prognostic factors in liver cancer patients. On this basis, we constructed a nomogram to predict the patient's probability of survival at one, two, and three years. The expression levels of four genes (*CTSV*, *CXCL8*, *MKI67* and *PRF1*) were included as indicators to establish the risk score (Fig. 6a). In addition, a calibration plot was drawn and indicated that our nomogram had good predictive performance (Fig. 6b–d).

### Validation of the PRG-related prognostic signature for HCC

**Fig. 7:**
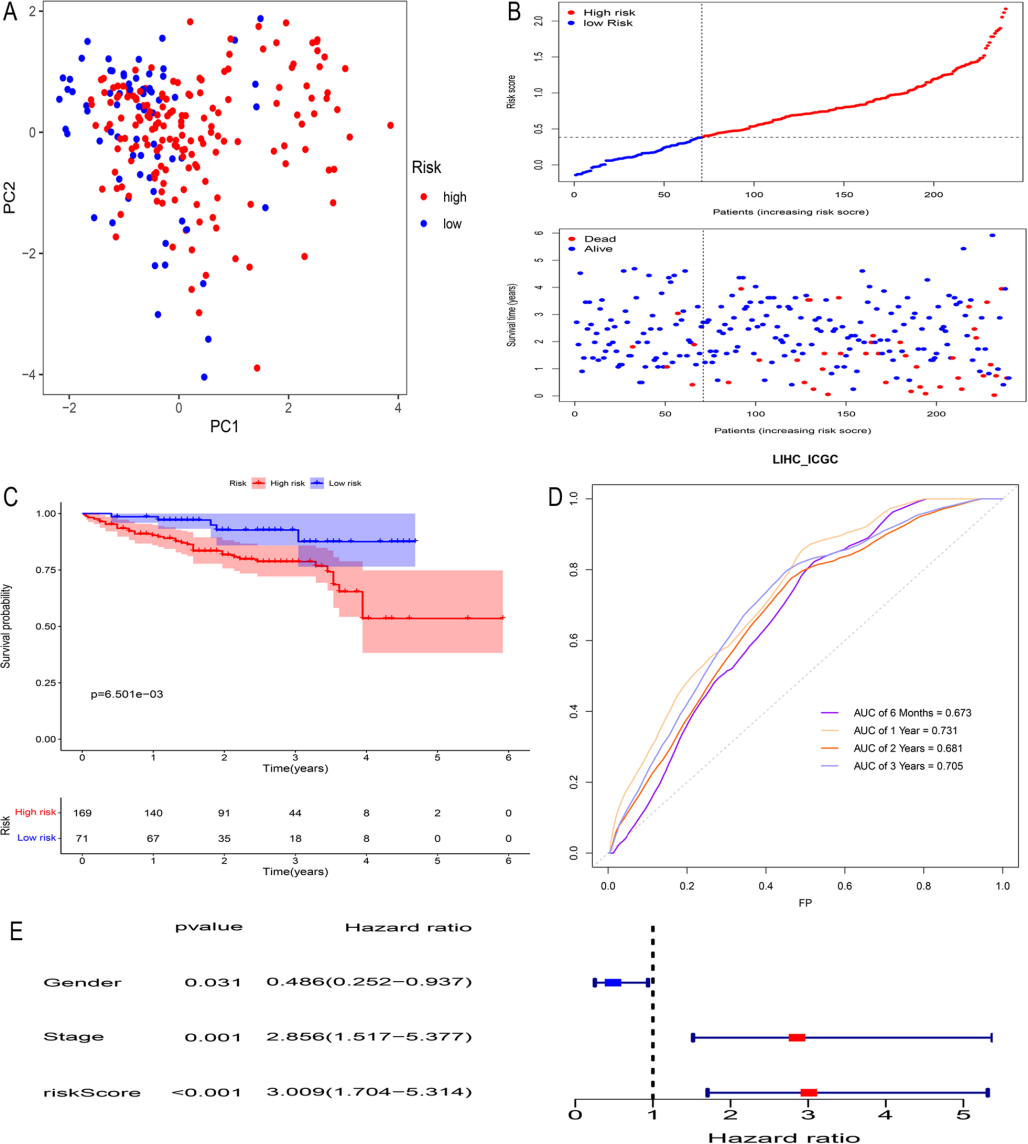
External validation of the ICGC cohort. PCA was performed by dividing HCC samples in the ICGC database into high- and low-risk groups using the cut-off values in TCGA (**a**). Samples with a high risk score in ICGC HCC samples had a worse prognosis (**b**, **c**). The t-ROC curve verifies the accuracy of the prognostic signature (**d**). Multivariate Cox regression showed that the risk score was an independent prognostic factor in the ICGC database (**e**)

To verify the availability of our PRG-related model, we downloaded liver cancer datasets from the ICGC database to verify the risk score equation. We used a cut-off value of 0.384 for the training set to divide the ICGC database samples into high- and low-risk subgroups. Consistent with the results from the TCGA database, patients in the high- and low-risk groups could still be distinguished by PCA (Fig. 7a). Patients with high risk scores had worse prognoses (Fig. 7b), and the OS of high-risk patients was significantly reduced compared with that of low-risk patients (*P* = 6.501e−03) (Fig. 7c). The AUCs of the prognostic signature at six months, one year, two years and three years were 0.673, 0.731, 0.681 and 0.705, respectively (Fig. 7d). Multivariate Cox regression proved that the prognostic signature was an independent prognostic factor for HCC (Fig. 7e). All of the above evidence indicates that PRGs are of certain clinical value in our prognostic signature of HCC.

### Gene enrichment analysis

**Fig. 8:**
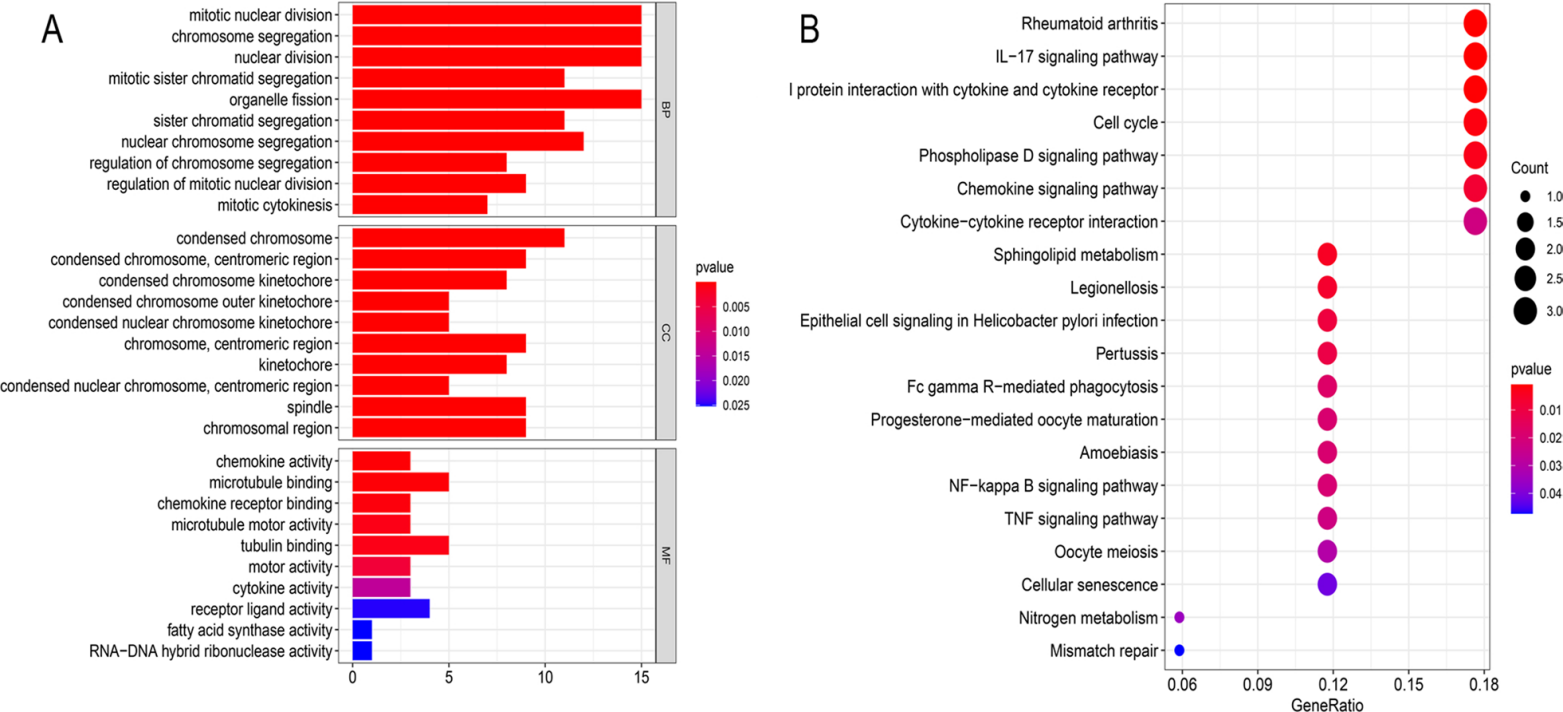
Functional analysis based on the DEGs between the two risk groups in the TCGA cohort. GO enrichment analysis, including biological process analysis, cellular component analysis and molecular function (**a**). KEGG pathway analysis (**b**)

To further explore the differences in gene function and pathways between the high- and low-risk subgroups, the "limma" package was used to identify DEGs between the two subgroups of TCGA patients. The differential criteria were an absolute value of log FC > 1 and *P* < 0.05. In total, 45 genes related to the risk score were found. Of these, 43 genes were upregulated in the high-risk group, and 2 were downregulated. Then, KEGG and GO enrichment analysis was conducted on these genes (Fig. 8a, b), and the results showed that most of the high risk-related pathways were cell cycle, cell metabolism and immune-related pathways.

### Analysis of the immune microenvironment in patient subgroups

**Fig. 9:**
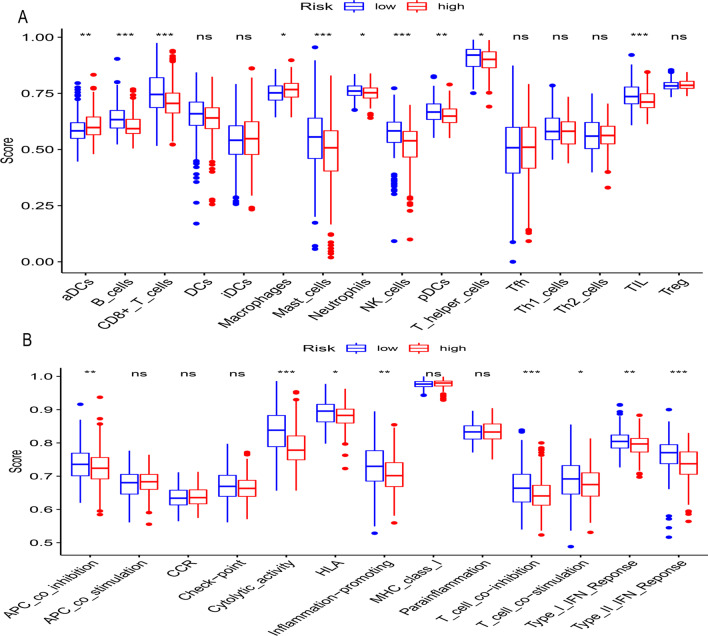
Analysis of the immune microenvironment in patient subgroups. Comparison of the enrichment scores of 16 types of immune cells and 13 immune-related pathways between the low- and high-risk groups in the TCGA cohort

To explore the differences in immune infiltration and the activity of immune-related pathways in high- to low-risk subgroups, single-sample gene enrichment analysis (ssGSEA) was used to calculate the immune score of 16 kinds of immune cells in each sample. The results showed that the high-risk group usually had a lower level of immune infiltration. In particular, B cells, CD8 + T cells, macrophages, mast cells, neutrophils, NK cells, pDCs (plasmacytoid dendritic cells), T helper cells, and TILs (tumour-infiltrating lymphocytes) (Fig. 9a) were found at lower levels in this group. The activity of immune-related pathways was also generally downregulated in the high-risk group (Fig. 9b).

## Discussion

HCC is a serious health hazard worldwide. Some studies have shown that pyroptosis plays an important role in the occurrence and development of tumours [21, 22], but the role of PRGs in liver cancer remains unclear. In this study, we first assessed 29 PRGs that were differentially expressed in tumour and normal samples and conducted PPI network and correlation analysis to explore the associations of these genes in HCC. To further explore the prognostic value of these PRGs, we established a risk profile of four genes by univariate Cox regression, LASSO-Cox regression, and multivariate Cox regression analyses, and the risk score equation was verified to be clinically significant in the ICGC database. A nomogram was constructed to estimate the patient's one-, two-, and three-year survival probability. Enrichment analysis showed that the DEGs between the high- and low-risk groups were involved in immune-related pathways. We conducted ssGSEA and found that the level of immune infiltration and the activity of related pathways were decreased significantly in the high-risk group.

Among the four key PRGs obtained, cathepsin V (*CTSV*) promotes the proliferation and invasion of tumour cells in breast cancer [23] and colorectal cancer [24], but its role in liver cancer is unclear. *CXCL8/IL-8* is a well-known chemokine that mediates cancer cell motility, invasion and metastasis by promoting epithelial-mesenchymal transition (*EMT*) [25] while its role in HCC has been little explored. IL-8 has been found to increase cancer proliferation in vitro [26]. In HCC, the *IL-8* gene expression can be regulated by transcriptional activation of NF-κB, activation of the ERK, p38 mitogen-activated protein kinase (MAPK) and PI3K pathway [27]. Furthermore, NF-κB activation has been proven to be related to gasdermin D which is the executor of pyroptosis [28]. In Zhao et al.'s study [29], *MKI67* was confirmed to be related to the prognosis of liver cancer. Upregulation of MKI67 elevates the degree of immune infiltration of many immune cell subtypes within LIHC, including functional T cells, CD4 + T cells, and CD8 + T cells [30]. However, the molecular mechanism of its involvement in pyrotopia in hepatocellular carcinoma is still unclear. Perforin, a pore-forming protein encoded by the *PRF1* gene [31] and plays a crucial role in the killer cell-mediated elimination of virally infected host cells, tumour cells [32]. Perforin plays a dominant role in the CD8 + T cell-mediated lysis of HCV-replicating human hepatoma cells [33]. At the same time, in HBV-specific hepatocellular carcinoma, perforin also plays a certain cytotoxic role [34]. Our results indicate that these four key genes are independent factors affecting the prognosis of HCC, and the signature including all these genes has good predictive performance in terms of prognosis and recurrence.

There are some shortcomings in our present study. Our signature should be validated further by performing clinical trials to better evaluate the relationship between the risk-score and pyroptosis in HCC. Besides, specific molecular mechanisms of 4 PRGs in our signature needs further examination.

In conclusion, our study suggests that the prognosis of HCC is closely related to pyroptosis. We constructed a promising PRG prognostic model to predict the prognosis of HCC patients. It will be a helpful reference for clinical and treatment decision-making. At present, there are few studies on pyroptosis, especially its mechanism in HCC. The four genes in the signature may play an important role in pyroptosis in HCC. Our study preliminarily confirmed the prognostic value of these PRGs in HCC.

## Conclusion

In summary, we identified pyroptosis-related DEGs between HCC and normal tissues and provided a signature to evaluate the prognosis of HCC based on pyroptosis-related genes.

## Supplementary Information

**Additional file 1.** CC analysis to explore patient differences.

## Data Availability

The datasets analysed in this research were downloaded from The Cancer Genome Atlas (TCGA, https://portal.gdc.cancer.gov/repository), the UCSC Xena platform (https://xenabrowser.net/) and the International Cancer Genome Consortium (ICGC, https://dcc.icgc.org/releases/current/Projects/LIRI-JP).

## Abbreviations

HCC: Hepatocellular carcinoma
RCD: Pyroptosis is a form of regulated cell death
PRG: Pyroptosis-related genes
mRNA-seq: MRNA expression profiles
TCGA: The Cancer Genome Atlas
ICGC: International Cancer Genome Consortium
CC: Consensus clustering
DEG: Differentially expressed gene
LASSO: Least absolute shrinkage and selection operator
OS: Overall survival
KM: Kaplan–Meier
t-ROC: Time-dependent receiver operating characteristic
PCA: Principal component analysis
TIMER: Tumour Immune Estimation Resource
HR: Hazard ratio
AUC: Area under the ROC curve
CI: Confidence interval
KEGG: Kyoto Encyclopedia of Genes and Genomes
GO: Gene ontology
PPI: Protein-protein interaction
ssGSEA: Single-sample gene enrichment analysis
