# Supplementary material for: Prognostic signature for hepatocellular carcinoma based on 4 pyroptosis-related genes
Source: BMC Med Genomics. 2022 Jul 28;15:166. doi: 10.1186/s12920-022-01322-9 (PMC9336086; doi:10.1186/s12920-022-01322-9)
Supplement: Supplementary file 1 — Additional file 1. CC analysis to explore patient differences. [file 12920_2022_1322_MOESM1_ESM.docx]

**
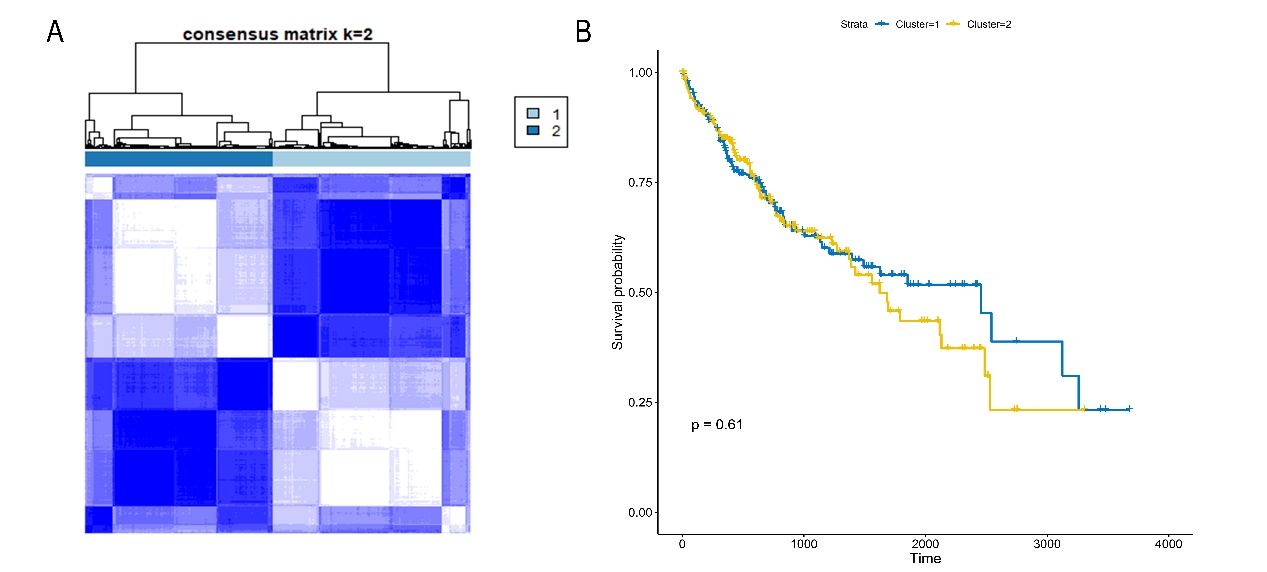
**

**CC analysis to explore patient differences**

In total, 367 HCC patients were grouped into two clusters according to the consensus clustering matrix (k = 2) (a). KM curves for the two clusters found no significant differences between the two clusters (b).
